# Prognostic factors in thyroid carcinomas: a 17-year outcome study

**DOI:** 10.20945/2359-3997000000175

**Published:** 2019-09-25

**Authors:** Tanja Makazlieva, Olivija Vaskova, Sinisha Stojanoski, Manevska Nevena, Daniela Miladinova, Vesna Velikj Stefanovska

**Affiliations:** 1 Institute of Pathophysiology and Nuclear Medicine Medical Faculty University Ss Cyril and Methodius Skopje Republic of North Macedonia Institute of Pathophysiology and Nuclear Medicine, Medical Faculty, University Ss Cyril and Methodius, Skopje, Republic of North Macedonia; 2 Institute of Epidemiology and Statistics with Medical Informatics University Ss Cyril and Methodius Skopje Republic of North Macedonia Institute of Epidemiology and Statistics with Medical Informatics, University Ss Cyril and Methodius, Skopje, Republic of North Macedonia

**Keywords:** Thyroid carcinomas, survival rate, prognostic factors

## Abstract

**Objectives:**

The aim of our study was to evaluate the survival rate of all thyroid carcinomas (TCs) diagnosed in the 1999–2015 period in the Republic of North Macedonia and to analyze the prognostic influence of several characteristics on development of distant metastases, as well as to analyze the prognostic effect of seven clinical and constitutional features on mortality.

**Material and methods:**

A retrospective analysis of medical data from all TCs diagnosed in 1999–2015 was performed. The survival rate of all types of TCs was estimated using the Kaplan Meier method. Univariate and multivariate logistic regression analysis was applied for evaluation of the predictive role of seven clinical and constitutional characteristics for development of distant metastases, and the univariate Cox-proportional model was applied for evaluation of the predictors for mortality.

**Results:**

A total of 422 TC cases were diagnosed in the 17-year period, with an average survival time of 212.99 months. Results of the univariate regression analysis showed that dimension at initial ultrasound and histopathological type of tumor were significantly predictive variables for distant metastases. Multifocal tumors vs. unifocal tumors < 15 mm significantly increased the probability of distant metastases by 7.401 (p = 0.005, 95% CI = 1.817–30.190) times. Age, initial lymph node involvement, number of radioiodine therapies, and histopathology of the tumor were selected as independent significant predictors for mortality.

**Conclusion:**

Our results showed an excellent overall prognosis of thyroid tumors in the Macedonian population. The dimension of the tumor, multifocality, and histopathological type were the most relevant prognostic predictive features for development of distant metastases.

## INTRODUCTION

Thyroid carcinomas (TCs) usually arise from follicular epithelial cells. Less frequent are medullary TCs of neuroendocrine origin, and extremely rare are thyroid tumors of mesenchymal origin (1). Further classification taking into consideration genetic profiling is necessary (2). According to histopathological findings, there are three main groups of TCs: well-differentiated (DTCs), poorly differentiated (PDTCs), and undifferentiated or anaplastic TCs (ATCs). DTCs are most prevalent, with papillary thyroid carcinoma (PTC) being the most frequent, followed by follicular thyroid carcinoma (FTC). Great diversity exists even in the PTC group, consisting of several subtypes according to distinct histopathological features, genetic molecular findings, aggressiveness, and prognosis (1-6). The most frequent are typical and follicular variants, but the most adverse are tall cell and diffuse sclerosing variants of PTC (5-8). FTC is the second most common TC, and according to the WHO classification is characterized by relatively well-differentiated cancer cells and absence of typical nuclear features of PTC (9-13).

Since 2004, based on their features, PDTCs have been introduced into the WHO classification as a separate category (between DTCs and ATCs) (14,15). Undifferentiated or ATC is a rare form, represented by 1–2% of TCs, but characterized by aggressive biology. It accounts for 14–50% of all deaths in TC patients, with an average survival rate of 3–5 months, despite the use of available multimodality therapy (16). Clinical and constitutional findings are important for the initial risk stratification and prognosis, but dynamic risk stratification and reevaluation of the prognostic factors are also important during the follow-up period (17,18).

The objective of our study was to evaluate the survival rate of all TCs diagnosed in Republic of North Macedonia during the 1999–2015 period and to analyze the prognostic influence of seven parameters on development of distant metastases during the follow-up period, as well as to analyze the prognostic effect of seven clinical and constitutional features on mortality.

## MATERIALS AND METHODS

A retrospective analysis was performed of the medical data of all TCs in the 1999–2015 period, diagnosed at the Institute of Pathophysiology and Nuclear Medicine in Skopje, as well as the Nuclear Medicine department at the Clinical Hospital in Bitola. These departments were the two main centers treating TC patients in our country during the evaluated period; hence, we believe that in the absence of a regional cancer registry, the presented number of TCs is the most precise and representative for the whole country. The two centers used the same therapy protocol and total and near total thyroidectomy was mostly applied in all differentiated TCs larger than 1 cm; iodine ablation and radioiodine therapies were performed according appropriate protocols (10). ATCs were further treated at the Oncology department, and medullary TCs were only surgically treated. Therapy with tyrosine kinase inhibitors was not available in our country for the evaluated period. We divided TC cases into five groups: (1) Carcinoma anaplasticum (ATC); (2) Carcinoma folliculare (FTC), including Hurthle cell carcinoma; (3) Carcinoma papillare (PTC); (4) Carcinoma medullare (MTC); and (5) mixed group of rare types of thyroid tumors (RTTs), such as tumors from mesenchymal origin lymphomas, sarcomas, and metastatic tumors in the thyroid. Cumulative proportional survival rate was analyzed using the Kaplan-Meier method for the entire sample of TCs and separately for the five distinct types.

Statistical analysis was performed using SPSS, v.21.0 for Windows. Data are expressed as percentages, mean, and standard deviation. Univariate and multivariate logistic regression analysis was performed for evaluation of the possible predictive role of seven clinical and constitutional characteristics for development of distant metastases. We evaluated the possible predictive role of gender, age, familial anamnesis, echogenicity of the primary tumor, dimension of the tumor at initial ultrasound (US), preoperative thyroid hormonal status, and histopathological type of the tumor.

The univariate Cox-proportional model was applied for evaluation of the independent predictors for mortality. Seven clinical and constitutional variables were included: age at initial diagnosis, gender, presence of lymph node involvement at initial presentation, number of applied radioiodine therapies for differentiated TCs, total received dose of radioiodine only for differentiated TCs, period from surgery to radioiodine ablation, and histopathology type of the tumor. The level of significance was *P* < 0.05.

## RESULTS

A total of 422 TC cases were diagnosed and treated in the 17-year period (1999–2015) at two main thyroid departments. From the total of 422 cases, histopathological findings and precise histopathological diagnosis of the tumor were well documented in 386 (91.47%) cases, the most common being PTC with 307 cases (79.5%), followed by FTC with 42 cases (10.9%), MTC with 16 (4.1%), ATC with 12 (3.1%), and other RTT with 9 (2.3%) cases. We found appropriate data for evaluation of distant metastases in half of the TC patients (218/422; 51.66%), of which 70 (32.11%) had metastatic disease (Table 1).

**Table 1:** Analysis of TC patients according to developed metastases

Metastatic status	Number	%
Without	148	67.88
Undefined	10	4.6
Skeletal	2	0.92
Lung	25	11.47
Skeletal and lung	2	0.92
Parotid gland	3	1.38
Neck lymph nodes	25	11.47
Local recurrence	3	1.38
Total	218	100

Results of the univariate logistic regression analysis showed dimension of the tumor at initial US examination and histopathological type of tumor to be significant predictive variables for distant metastases (Table 2). We found that a tumor dimension of 16–50 mm *vs.* tumor < 15 mm (*p* < 0.05) significantly increased the probability of distant metastases by 6.154 (*p* = 0.004, 95% CI = 1.762–21.489) times. Multifocal tumors *vs*. tumors < 15 mm (*p* < 0.05) significantly increased the probability of distant metastases by 7.401 (*p* = 0.005, 95% CI = 1.817–30.190) times (Table 2).

**Table 2:** Predictive role of seven variables and probability for distant metastases, univariate logistic regression analysis

Variable	B	S.E.	Wald	df	Sig.	Exp(B)	95% C.I. for EXP(B)	

							Lower	Upper
Gender – reference category/male								
Female	(0.655)	0.349	3.516	1	0.061	0.520	0.262	1.030
Age - reference category/≤45 years								
>45 years	(0.018)	0.291	0.004	1	0.951	0.982	0.555	1.738
Dimension of the tumor on US/< 15 mm								
16-50 mm	1.817	0.638	8.111	1	0.004*	6.154	1.762	21.489
>51 mm	2.708	0.771	12.337	1	0.000*	15.000	3.310	67.978
Multifocal	2.002	0.717	7.803	1	0.005*	7.407	1.817	30.190
Echogenicity – reference category/hyperechogenic								
Anechogenic	(19.006)	14210.361	0.000	1	0.999	0.000	0.000	.
Hypoechogenic	1.759	1.092	2.593	1	0.107	5.806	0.682	49.406
Isoechogenic	2.197	1.269	2.997	1	0.083	9.000	0.748	108.310
Inhomogeneous	1.099	1.089	1.018	1	0.313	3.000	0.355	25.339
Familial anamnesis – reference category/no								
Positive	(0.376)	0.375	1.003	1	0.316	0.687	0.329	1.433
Hormonal status – reference category/euthyroid								
Hypothyroid	(20.321)	40192.970	0.000	1	1.000	0.000	0.000	.
Hyperthyroid	0.323	0.657	0.241	1	0.623	1.381	0.381	5.009
Subclinical hyper.	0.372	0.757	0.241	1	0.623	1.450	0.329	6.390
Subclinical hypo.	(0.322)	0.687	0.219	1	0.640	0.725	0.188	2.789
Histopathological type - reference category/PTC								
FTC	0.943	0.399	5.600	1	0.018*	2.568	1.176	5.608
MTC	(20.195)	17974.843	0.000	1	0.999	0.000	0.000	.
RTT	22.211	40192.970	0.000	1	1.000	44.644	0.000	.
ATC	2.617	1.109	5.570	1	0.018*	13.696	1.558	120.361

Dependent variable – distant metastases. * Significant for p < 0.05.

Analysis of histopathology results showed that FTC *vs.* PTC (*p* < 0.05) significantly increased the probability of distant metastases by 2.568 (*p* = 0.018, 95% CI = 1.176–5.608) times (Table 2).

All variables selected through univariate logistic regression analysis as significant predictors for development of distant metastases were analyzed using multiple logistic regression analysis (Table 3). The results obtained with this method confirmed the following independent predictors for metastatic disease: dimension of the tumor (16–50 mm, > 51 mm and multifocal) compared to tumors < 15 mm and histopathological type ATC *vs.* PTC (Table 3).

**Table 3:** Multiple logistic regression analysis of predictors for distant metastases

Variable	B	S.E.	Wald	Df	Sig.	Exp(B)	95% C.I. for EXP(B)	

							Lower	Upper
Dimension of the tumor on US/< 15 mm								
16-50 mm	1.492	0.650	5.268	1	0.022*	4.447	1.244	15.905
> 51 mm	2.637	0.802	10.817	1	0.001*	13.966	2.902	67.210
Multifocal	1.984	0.718	7.626	1	0.006*	7.271	1.779	29.723
Histopathological type – reference category/ PTC								
FTC	0.527	0.503	1.098	1	0.295	1.694	0.632	4.544
MTC	(20.269)	19039.427	0.000	1	0.999	0.000	0.000	.
RTT	22.211	40192.970	0.000	1	1.000	44.644	0.000	.
ATC	2.729	1.127	5.865	1	.015*	15.310	1.682	139.324

Dependent variable – distant metastases. * Significant for p < 0.05.

**Figure 1:**
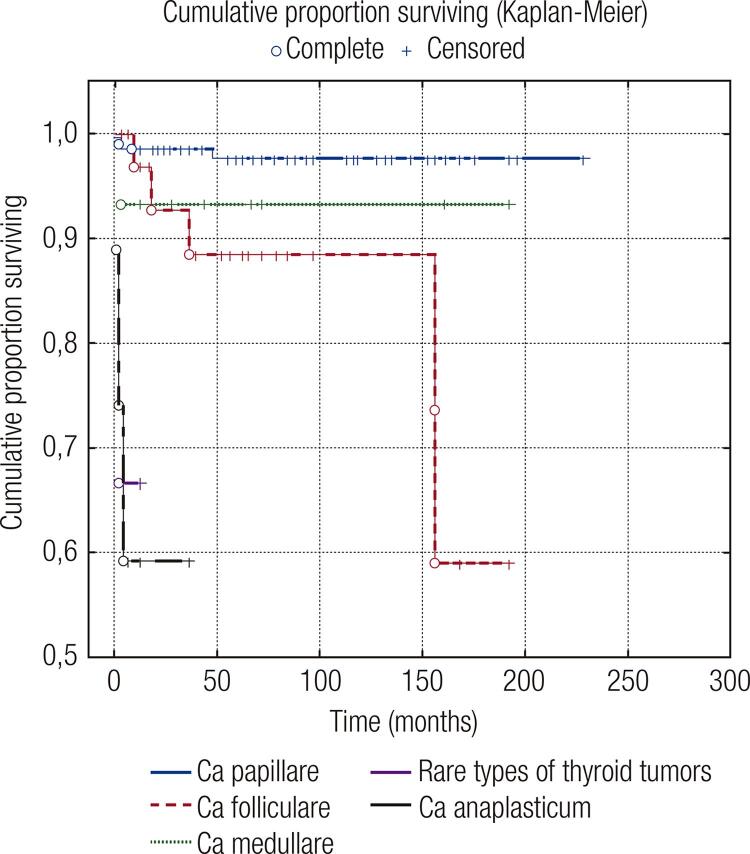
Overall survival in patients with TCs according to histopathological types.

The mean survival time of all TC patients was 212.99 (95% CI = 204.6–221.4) months (Figure 1). The mean survival time by histopathological type was as follows: for PTC, 223.07 (95% CI = 218.6–227.5) months; for FTC, 161 (95% CI = 138.8–184.7) months; for MTC, 179 (95% CI = 155.5–203.3) months; for RTT, 8.7 (95% CI = 3.3–14.0) months; and for ATC, 22.3 (95% CI = 10.2–34.4) months. There was a significant difference between different histopathological types (*p* < 0.05) (log-rank/Mantel-Cox: chi-square = 62.245; df = 4; *p* < 0.0001).

The worst prognosis was observed in the mixed group consisting of other RTTs, with a survival time longer than 10 months in 67% of patients, while only 58% of ATC patients survived more than 25 months (Table 4).

**Table 4:** Analysis of survival rate according to different histopathological types of TC

Histopathological types of TC	Mean			

	Estimation (months)	Std. Error	95% confidence interval	

			Lower bound	Upper bound
PTC	223.068	2.255	218.648	227.488
FTC	161.768	11.713	138.810	184.726
MTC	179.400	12.173	155.541	203.259
RTT	8.667	2.722	3.332	14.001
ATC	22.333	6.183	10.214	34.453
For all types	212.993	4.267	204.630	221.356

Four parameters (age, lymph node involvement at initial presentation, number of radioiodine therapies, and histopathological type of the tumor) were selected through the Cox-proportional model as independent significant predictors for mortality (Table 5). Age > 45 significantly increased likelihood of death events by 42.9%/month (Cox-proportional model: Exp (B) (HR) = 8.429 [*p* = 0.005, 95% CI = 1.933–36.759]) (*p* < 0.05). Neck lymph node metastases at initial examination of the patients also significantly increased probability of death events by 46.7%/months (Cox-proportional model: Exp (B) (HR) = 3.467 [*p* = 0.009, 95% CI = 1.368–8.787]) (Table 5).

**Table 5:** Univariate Cox Proportional model in evaluation of seven parameters as predictors for mortality

Variables	B	S.E.	Wald	Df	Sig.	Exp(B)	95% C.I. for EXP(B)	

							Lower	Upper
Gender – reference category/ male								
Female	(.234)	0.567	0.170	1	0.680	0.791	0.260	2.406
Age - reference category / ≤45 years								
>45 years	2.132	0.751	8.048	1	0.005*	8.429	1.933	36.759
Initial lymph node involvement- reference category / absent								
Present	1.243	0.474	6.870	1	0.009*	3.467	1.368	8.787
Number of radioiodine therapies (RAI)								
Number of RAI	0.257	0.063	16.563	1	0.000*	1.293	1.142	1.463
Total dose of RAI therapy								
Total dose	(0.001)	0.003	0.165	1	0.685	0.999	0.993	1.005
Period from operation till radioiodine ablative therapy								
Months	(0.067)	0.103	0.429	1	0.513	0.935	0.765	1.143
Histopathological type of TC - reference category / Ca papillare								
FTC	1.866	0.638	8.557	1	0.003*	6.462	1.851	22.557
MTC	1.418	1.097	1.669	1	0.196	4.127	0.480	35.458
RTT	3.720	1.128	10.885	1	0.0001*	41.267	4.527	376.172
ATC	3.694	0.755	23.964	1	0.000*	40.224	9.164	176.559

Dependent variable – alive/dead. * Significant for p < 0.05.

## DISCUSSION

Our analysis revealed 422 cases of TCs in the period of 17 years in the total population of 2,020,157, or a 1.22/10^5^ prevalence rate of TCs, in the Republic of North Macedonia. We evaluated seven parameters to understand the possible implications in their predictive role for development of distant metastases and mortality of TCs in the Macedonian population over the analyzed period. The average survival time for all TCs for the Macedonian population was 212.99 months, with the longest average survival time for PTC. Ninety-eight percent of patients with PTC survived longer than 16 years; similar findings were presented in the study of Ito Y and cols. (19,20). On the other hand, only 59% of FTC patients survived longer than 13 years; the average survival time for FTC patients was 161 months. Ninety-four percent of patients with MTC survived longer than 12 years, which is similar to the findings in the literature (10,21-24). Differences were detected in the survival of ATC group of patients, because our results showed a longer average survival of 22.3 months (95% CI = 10.2–34.4), compared to the average survival of 6 months reported in the literature (10). In the study of Nachalon Y and cols., the outcome of ATC patients varied greatly depending on the type of selected treatment, whether it was radiotherapy, chemotherapy protocol, palliative surgery alone, or combined therapy (10,25).

Our results from multiple logistic regression analyses revealed a significant association of dimension and histopathological type of tumor as independent predictors for appearance of metastases. Tumors with a dimension of 15–50 mm, > 51 mm, and multifocal tumors were more often associated with distant metastases during the follow-up period compared to tumors smaller than 15 mm at initial US examination, underlining the importance of early diagnosis. This finding suggest that the US-guided fine needle aspiration biopsy (FNAB) should be considered even in smaller lesions (less than 10 mm) when there is a suspicious US, because this could lead to earlier diagnosis and better prognosis (26).

Our research revealed that multifocal PTCs are frequent and were associated with more aggressive nature of the disease and with metastatic disease, compared to tumors smaller than 15 mm. One study analyzed the molecular characteristics of multifocal PTCs in 17 patients with RET/PTC rearrangements, and in 15 cases, different types of this genetic rearrangement were identified, suggesting that they were different tumors independently developed in the organism with genetic predisposition and appropriate external influences (27). A recent meta-analysis comprising 21 articles showed that multifocality was associated with an increased risk of lymph node involvement, extrathyroidal extension, and disease recurrence (28). Another study also found that multifocality and increasing number of tumor foci were associated with disease recurrence, with more aggressive features and poorer prognostic outcome (29). Multifocality was the most common feature in PTCs, leading to the conclusion that total thyroidectomy might be more appropriate in this type of tumor due to the possibility of small foci of disease present in the contralateral thyroid lobe, thus lowering the possibility of locoregional recurrence of the disease.

The second independent predictor for distant metastasis was histopathological type of the thyroid tumor. In the subgroup of DTC, FTC was more frequently associated with metastatic disease 2.568-fold in comparison with PTC. This is important because FNAB is more prone to erroneous and inappropriate diagnosis of FTC because very often, a small capsular and vascular invasion might not be detected in the FNAB sample. Lee EK and cols. analyzed the serum thyroglobulin (sTg) level as a potential biomarker in differentiation among FTC and benign thyroid follicular lesions, and according to their study, preoperative Tg levels had high specificity in predicting thyroid cancer in the case of suspicious follicular neoplasm, suggesting possible usefulness as a marker in the cytological diagnosis of indeterminate nodules (30).

Clinical experiences showed that the dimension of the tumor is not always correlated with biological aggressiveness and prognosis in DTC (31,32). These observations and advances in molecular techniques imposed the need for new TC classification, and in addition to histopathological evaluation, introducing molecular genetic profiling should be considered in reclassifying TCs and preparing for individualized therapeutic maneuvers adjusted according to specified genetic mutation profiles (33,34).

Prognostication of thyroid tumors can be made at initial presentation with several different, but similar, prognostic scoring systems. For follicular cell-derived tumors, the AGES (age, histologic grade of the tumor, extent of extrathyroidal invasion or distant metastases, and size of the primary tumor) score system, AMES (patient age, presence of distant metastases, extent and size of the primary tumor) score, MACIS (metastasis, patient age, completeness of resection, local invasion, and tumor size) score, and the TNM (tumor, node, metastasis) system were introduced (35).

Few risk stratification systems have been introduced for scoring the disease, such as AMES, MACIS, and American Joint Committee on Cancer (AJCC) TNM classifications (10). These classification systems enable initial risk stratification of the patients, but Tuttel and cols. and Momesso and cols. also suggested ongoing, dynamic risk stratification during follow-up of the patients (18,36,37). Age is one constitutional factor that is considered in many stratification systems for TCs. Differentiated TC patients under a certain age cutoff are considered to be at less risk than those who are older. For example, age 55 was the cutoff point for continuous variables in the Memorial Sloan Kettering (grade, age, metastasis, extent, size, or GAMES) and AJCC/UICC systems (8th edition) (37), but there is a lack of consensus among the staging systems regarding the age threshold that has to be adopted (38).

We evaluated the prognostic influence of seven independent characteristics on the outcome of the disease, and four characteristics, according to the Cox-proportional model, were revealed as significant prognostic predictors for survival: age, lymph node involvement at initial presentation, number of radioiodine therapies, and histopathological type of thyroid tumor. In our study, age > 45 years compared to ≤ 45 years, as well as histopathological type FTC *vs.* PTC, significantly (*p* < 0.05) increased the probability of a death event. Another feature evaluated in our study was the need for an increased number of radioiodine therapies, which pointed out an unfavorable prognosis in PTC and FTC, probably due to dedifferentiation of the TCs. Due to enlargement of the tumor for one evaluated group, the radioiodine therapy was increased by 15.038 mCi.

Approximately 80% of patients with DTC are cured after initial therapy and have excellent prognosis (10, 18). Mazzaferri and cols. performed a similar analysis in the Italian population, and of 213 patients with DTC, 75% reached complete response to treatment after 12 months. The same group of authors analyzed the prognostic relevance of a few factors on survival rate in 1510 patients, without initial distant metastases. They concluded that the probability of a death event increased with age above 40 years, size of the tumor > 1 cm, local tumor invasion and initial neck lymph node involvement, FTC histopathology, and delay in treatment. Their findings are similar to the results obtained in our study. These data point out the importance of careful initial risk stratification and appropriate initial treatment, as well as wise selection of the methods used in treatment in terms of avoiding overtreatment or incomplete therapy at the start (27).

The prognostic significance of initial neck lymph node involvement in DTC is still controversial. Several studies found an increased risk for local recurrence in patients with initial lymph node involvement, and our study revealed a significant correlation between the initial metastatic disease in neck lymph nodes and total radioiodine therapy received by patients and more frequent recurrence in this group of patients (31,32,39). Due to these findings, there is still an ongoing debate about the extent of surgical treatment with respect to neck lymphadenectomy. According to contemporary guidelines, there are two possibilities: bilateral central neck lymphadenectomy and/or modified lateral neck compartment dissection, only if enlarged lymph nodes have been detected preoperatively or intraoperatively (7,18,28). According to our findings, a careful preoperative neck examination is necessary for initial risk assignment and for selection of an appropriate surgical approach.

The limitations in our study were the heterogeneity of the thyroid tumors included in the analyses, considering the different types of TCs together, as well as possible influence of different therapy protocols applied. However, the limitations of this research point toward topics to be addressed in the future.

In conclusion, our results showed an excellent overall prognosis of thyroid tumors in the Macedonian population. From seven clinical and constitutional features, dimension and histopathological type of thyroid tumors were the most relevant prognostic predictive features for development of distant metastases in the evaluated group of patients. Our findings suggest that diagnosis of small primary tumors is important in improving survival rate, and detection of multifocality and neck lymph node involvement should be considered as increasing risk stratification factors. These characteristics, according to our analyses, could be important because they could have a significant impact on selecting the appropriate type of surgical treatment. Further studies including analysis of different therapy approaches on the outcome of TCs, as well as studies evaluating prognostic factors separately for different histopathological types of TCs, are needed for completing the evaluation of our group of patients.
